# Leech (Hirudo medicinalis) Therapy for the Treatment of Nipple-Areolar Complex Congestion Following Breast Reduction

**Published:** 2015-07-31

**Authors:** Matthew Freeman, Martin Carney, Tim Matatov, Rahul Vemula, Christopher Babycos

**Affiliations:** ^a^Department of Surgery, Division of Plastic and Reconstructive Surgery, Tulane University School of Medicine, New Orleans, La; ^b^Ochsner Clinic Foundation, New Orleans, La

**Keywords:** breast reduction, mammoplasty, nipple congestion, leech therapy, macromastia

**Figure:**
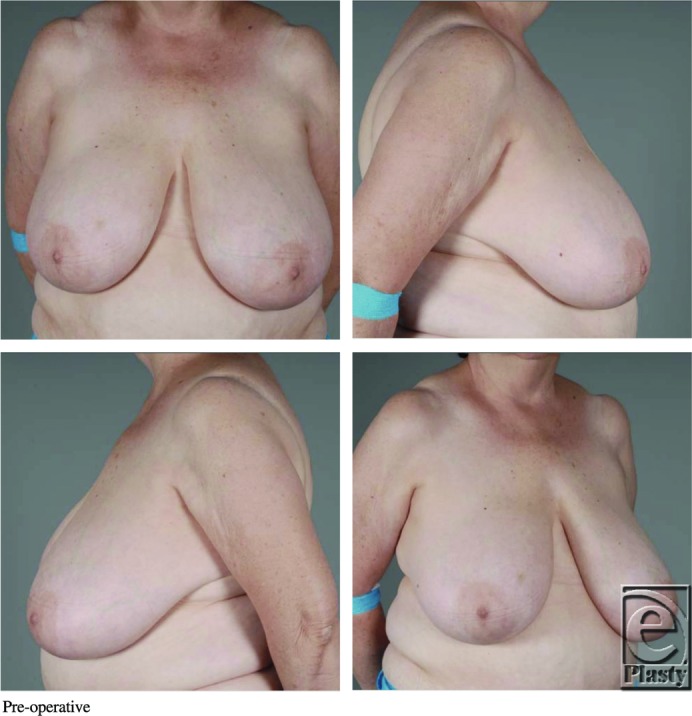


**Figure:**
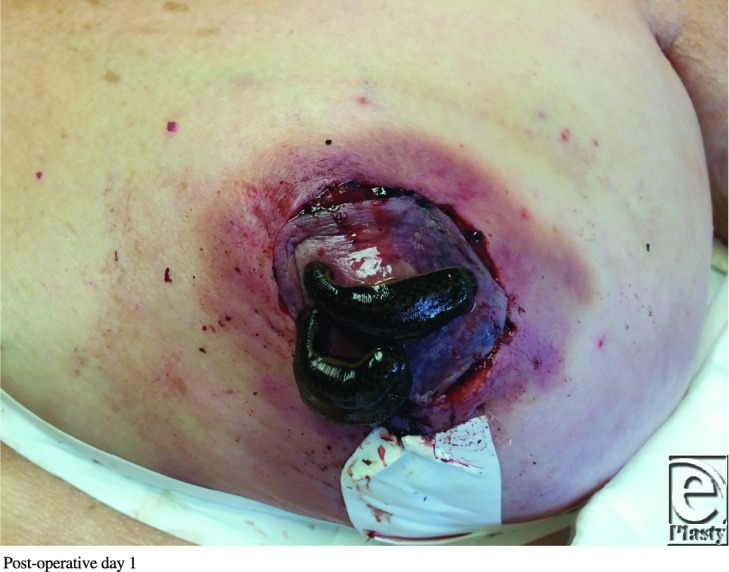


**Figure:**
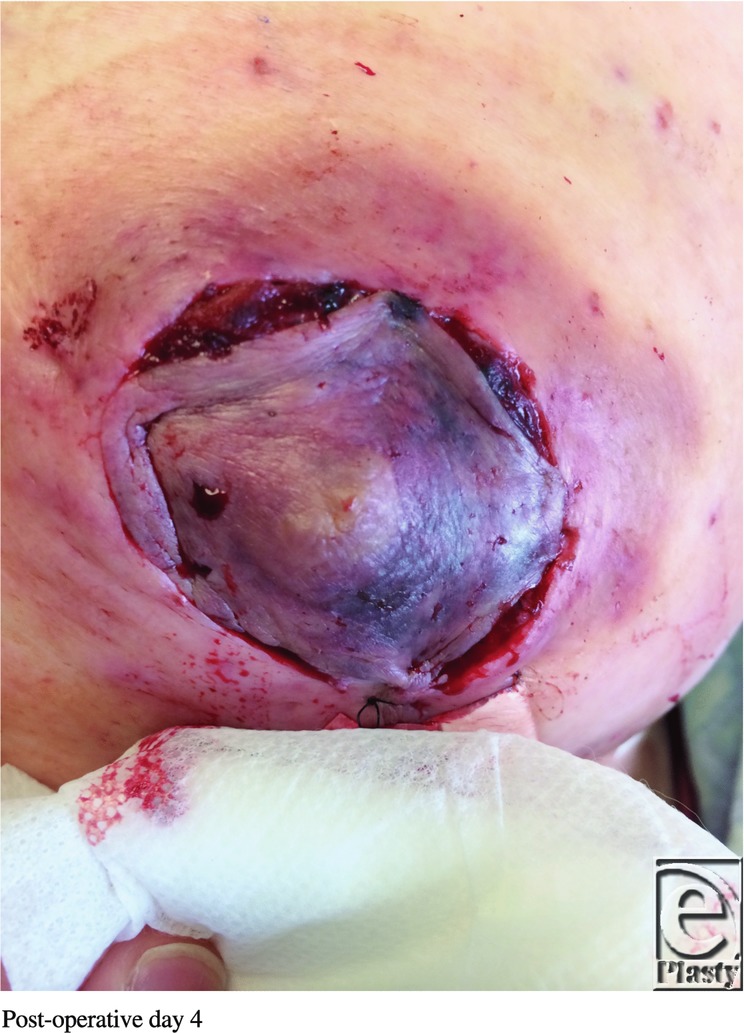


**Figure:**
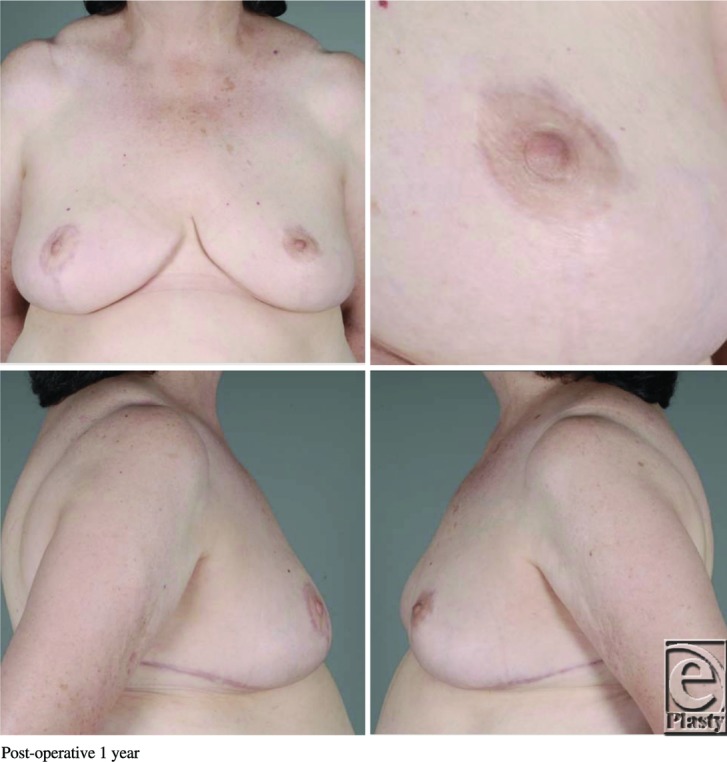


## DESCRIPTION

A 61-year-old obese woman presented to the plastic surgery clinic with macromastia. She underwent a Wise pattern reduction mammoplasty using an inferior pedicle without incident. On postoperative day 1, her left nipple-areolar complex (NAC) displayed venous congestion without evidence of hematoma.

## QUESTIONS

1. **What is the pathophysiology of NAC congestion following reduction mammoplasty?**
2. **What are the most common treatment modalities for NAC congestion?**
3. **What is the proper treatment technique and schedule when utilizing leech therapy?**
4. **How can potential complications of leech therapy be avoided?**

## DISCUSSION

Although more common than arterial insufficiency, venous congestion of the NAC during breast reduction surgery is a relatively uncommon complication. Larger reduction weights have been associated with higher rates of venous congestion.^1^ Causes of congestion include inadequate preservation of venous drainage, pedicle constriction secondary to tight inset, or hematoma formation. Furthermore, typical comorbidities such as smoking, diabetes, and obesity increase risk for venous congestion.^1^ Venous congestion can lead to NAC necrosis or conversion to a free nipple graft, which reduces lactation and sensation viability. The superomedial/medial and inferior pedicle techniques have been shown in cadaveric studies to have the most reliable and consistent venous drainage.^2^ Treatment of NAC congestion starts with prevention, followed by assessment and optimization of intrinsic patient characteristics. Weight loss, smoking cessation, blood pressure optimization, and diabetic control all reduce the incidence of NAC congestion.^1^ A free nipple graft should be considered on patients with body mass index of more than 35 kg/m^2^ or a nipple to inframammary fold distance of more than 18 cm. If there is concern over NAC viability intraoperatively, the procedure can be converted to a free nipple graft. However, conservative measures should be attempted first. The surgeon should explore for a hematoma and release tight periareolar sutures. When noted postoperatively, nitropaste and leeches can be used for a struggling NAC before necessitating a return to the operating room.^3^^,^^4^ Leeches act as a bridge by allowing venous output until neovascularization occurs. Hyperbaric oxygen, systemic antibiotics, and moist dressings can also be used. Leech therapy was first seen in ancient Egyptian cave paintings dating around 1500 BC. It enjoyed popularity between 1825 and 1850 and now, over the past 15 years, is having another renaissance.^5^ Despite its long use, there is no universally agreed-upon schedule for leech therapy. Elyassi's protocol proposal recommends determining the number of leeches based on the size of the congested flap, with a rough estimate of 1 leech per 3 cm^2^ of congestion. Leeches should be changed every 2 hours, but tapered depending on the progress of the flap, with the recommendation to taper when there is 50% reduction in congestion. Upon detachment, the leeches should be euthanized in 70% ethanol. The site of attachment should be cleaned with hydrogen peroxide or heparinized saline.^6^ Some studies have used up to 6 leeches per treatment, whereas others have shown adequate results with just 1 leech.^6^ Therefore, close observation, photographs, capillary refill testing, and detailed handoffs at the bedside are crucial for determining the level of venous congestion.^6^ The major complications of hirudotherapy are psychological stress, induced anemia, and infection.^7^ Patients should be adequately informed and consented regarding leech therapy, and the patient should be assured of its medical merits. Anemia is a common occurrence due to continuous blood loss, and as high as 49.75% of patients require transfusion.^7^ Blood typing and crossmatching should occur before leech application. Hemoglobin and hematocrit should be checked before therapy and then again depending on the amount of leeches, the length of the treatment regimens, and the number of days of therapy.^6^ Infection is the most dangerous complication of leech therapy. *Aeromonas* species live in the leech gastrointestinal tract in a symbiotic relationship to help with blood digestion.^8^ Mishandling or squeezing of the leech may regurgitate *Aeromonas* into the patient. For this reason, patients should receive antibiotic prophylaxis throughout the regimen and receive post-therapy treatment for 24 hours. Fluoroquinolones seem to have the most consistent activity profile against *Aeromonas* species, but recent studies have shown multidrug-resistant strains that may warrant double coverage with a third/fourth-generation cephalosporin in the future.^6^

## References

[1] Gravante G, Araco A, Sorge R (2008). Postoperative wound infections after breast reductions: the role of smoking and the amount of tissue removed. Aesthetic Plast Surg.

[2] le Roux CM, Pan WR, Matousek SA, Ashton MW (2011). Preventing venous congestion of the nipple-areola complex: an anatomical guide to preserving essential venous drainage networks. Plast Reconstr Surg.

[3] Gross MP, Apesos J (1992). The use of leeches for treatment of venous congestion of the nipple following breast surgery. Aesthetic Plast Surg.

[4] Güneren E, Erolu L, Akba H (2000). The use of *Hirudo medicinalis* in nipple-areolar congestion. Ann Plast Surg.

[5] Whitaker IS, Rao J, Izadi D, Butler PE (2004). Historical Article: *Hirudo medicinalis*: ancient origins of, and trends in the use of medicinal leeches throughout history. Br J Oral Maxillofac Surg.

[6] Elyassi AR, Terres J, Rowshan HH (2013). Medicinal leech therapy on head and neck patients: a review of literature and proposed protocol. Oral Surg Oral Med Oral Pathol Oral Radiol.

[7] Whitaker IS, Oboumarzouk O, Rozen WM (2012). The efficacy of medicinal leeches in plastic and reconstructive surgery: a systematic review of 277 reported clinical cases. Microsurgery.

[8] Utley DS, Koch RJ, Goode RL (1998). The failing flap in facial plastic and reconstructive surgery: role of the medicinal leech. Laryngoscope.

